# Book Reviews

**Published:** 1809-01

**Authors:** 


### CRITICAL ANALYSIS
### OF THE
### RECENT PUBLICATIONS
### ON THE
### DIFFERENT BRANCHES OF PHYSIC, SURGERY, AND MEDICAL PHILOSOPHY.

---

*An Essay on Diseases incidental to Europeans in Hot Climates, with the Method of preventing their fatal Consequences.* By JAMES LIND, M. D. F. R. S. Ed. &c. *To which is added, an Appendix on Intermittent Fevers, and a simple and easy Way to render Sea-Water fresh, and to prevent the Scarcity of Provisions in long Voyages.* Sixth Edition. Richardson, &c. London.

THOUGH this performance has been long before the public, yet the importance of the subject, during the present state of the kingdom, will not permit us merely to announce a new edition. When our fleets and armies are stationed at, or moving to all parts of the world; a work, which gives a faithful description of the climate of each, and of the manner in which the lives of those who live only to protect us, are to be preserved, claims particular attention. The comprehensive view taken by the author is particularly valuable, inasmuch as most of his information is founded on actual observation, and improved by frequent repetitions and comparisons with the remarks of others.

" The recent examples, says our author, of the great mortality in hot climates, ought to draw the attention of all the commercial nations of Europe, towards the important object of preserving the health of their countrymen, whose business carries them beyond seas. Unhealthy settlements require a constant supply of people, and of course drain their mother-country of an incredible number, and some of those its most useful inhabitants. Of this the Spanish dominions abroad have furnished us with striking proofs: even at this day, many of the Spanish merchants and adventurers, who yearly take their departure from Europe, die at Porto-bello or Carthagena, soon after their landing.

" The Dutch settlements at Surinam, St. Eustatia, and Curaçoa, as well as several in the East Indies, have proved fatal in point of health to the Dutch; as have the islands of Martinico, St. Domingo, and lately the country of Guiana, to the French settlers.

" Great Britain itself has its *Jamaica;* where the number of English sacrificed to the climate is hardly credible, and only to be guessed at from the common computation, that this island formerly buried to the amount of the whole number of its white inhabitants once in five years; however, it has of late become more healthy.

" It is now a well known and certain truth, that of such Europeans peans as have fallen victims to the intemperature of foreign climates, nineteen in twenty have been cut off by fevers and fluxes: these being the prevailing and fatal diseases in unhealthy countries through all parts of the world.

"In my *Essay on preserving Seamen*, I have said, that a malignant fever of the remitting or intermitting kind, most frequently a double tertian, is the genuine produce of heat and moisture, is the autumnal fever of all hot countries, and is the epidemic disease between the tropics. To which I may add, that it is also the disease most fatal to Europeans in all hot and unhealthy climates."

The first division of the work is on the seasons of sickness and the diseases incidental to strangers in different parts of the world. This is, perhaps, the most important of the whole, because it may oftentimes be absolutely necessary to maintain garrisons at, and even to lay seige to, certain unhealthy forts; under which circumstances, it is of importance to know that at certain times a garrison may remain with impunity, or a fort be besieged without the certain destruction of the invading army. It is not less important if ever we should arrive at peace, for mercantile men to know that by properly governing their concerns, a trade to the most deleterious climate, and even the most unhealthy spot, may be conducted with safety as well as advantage. Too many of them are not aware of the danger which attends many parts of the European continent, and the Mediterranean Islands. On this account, the first section takes a short but comprehensive view of the diseases produced by unwholesome seasons in England, in the Netherlands, in Hungary, the Campania of Rome, the Island of Sardinia, Minorca, and Gibraltar. Respecting the latter, it must be admitted, when we consider the late disasters which attended that garrison and town, that the remarks of our author are unsatisfactory. We mean not to accuse Dr. Lind of inattention in this respect; he probably was never on that rock during a sickly season; and it is much to be regretted that we have hitherto had no satisfactory account of the fever which so suddenly arose and disappeared in that place.

A section follows on the climate of Canada, Newfoundland, Halifax, New England, Maryland, Virginia, South Carolina, Georgia, Florida, Mobile, Pensacola. The consideration of these having brought him nearer to the tropics, a chapter follows on the most unwholesome seasons, and the diseases in Africa. The first section comprehends Algiers, Tunis, Tripoli, Morocco, and Egypt. In these there is nothing but what is commonly known. A more minute account follows of the coast of Guinea, its soil, periodical rains, and heat; its healthy and sickly seasons; quantity and effects of its rains; surprising effects of the Harmatans; comparative degree of health in the different European settlements. Diseases and waters of the country, with remarks on the causes of the violence of diseases in that quarter of the globe. In this chapter we have an interesting note, which appears to be an observation of the author, as he quotes no other persons

"Some

" Some idea, says he, may be formed of the rude state of the interior parts of Guinea in general, from the account of this factory, related to me by a medical gentleman, who accompanied the first detachment of British troops which were sent to take possession of it. Their passage from Senegal being against the stream, was upwards of six weeks; during which time they lost in fevers more than a third of their number, and suffered exceedingly from the sultry heat and mosquittoes.

" On their arrival they found a small fort, built on an eminence, at a winding of the river: the country on both sides of the river was low, and covered with thick woods, except for the space of half a mile round the fort; and on one side it was inhabited by blacks, on the other by Arabs, who, during the dry season, wandered thither with their flocks for pasturage. Here all Nature seemed to be at enmity with man. They were prevented from walking in the woods by tygers, who were so daring, as often at night to attempt scaling the walls of the fort; and if by going armed, or in small parties, they should escape these, yet they were exposed to the bites of venomous serpents, of different kinds, and many of a most extraordinary size: the shores of the river swarmed with crocodiles: the earth had its white ants, the air its wild bees, its sand-flies, and its mosquittoes. These insects, though not the most tremendous, were perhaps their most distressing enemies. The ants devoured almost every article either of provision or apparel; scarce any precaution could elude their art; they raised a hollow cylinder of earth perpendicularly towards their object, and through it, as by a ladder, ascended in thousands: they were one of the greatest torments to the sick of that climate, and would reach the bed in a night's time, though hung at a distance from the ground; when their bites, like scalding water poured upon the skin, were more intolerable than the disease itself. The sand-flies and mosquittoes were exceeding numerous, and would have been esteemed severe plagues, had not one yet more severe occurred, the wild bees, who swarmed in such numbers, as to darken the air, and often hived in their rooms. Once or twice in a summer they were generally visited by a swarm of locusts, who came from the East like a thick cloud, and eat up every thing that was green; but this was only a temporary inconvenience, as in eight or ten days the earth was clothed with a new verdure, and the trees put forth new leaves."

" The whole animal creation here attained an unusual degree of perfection. Droves of elephants, and ostriches of a large size, frequently came down to the fort: the baboons were so numerous, that they made them their principal amusement; they clothed them with the regimentals of the soldiers who died, they made them walk erect, by tying their two fore-feet behind them, and in some respects even made them serve them in their houses.

" The sultry heat of the weather was almost intolerable, even the night could not be deemed cool, and, when the wind came from the the desert, it scorched like a blast from the mouth of an oven. Annually the country was subject to an inundation of the river, from the heavy rains which fall periodically in that climate: these continued for several months, and laid the country for a great extent under water; when the little fort, defended by its high walls, appeared like a small island in the midst of a sea. This was the season of sickness, and it swept off near one-half their number; during it, the slightest irregularity or intemperance was productive of death; a company would one night meet and be merry, and before the next, be the greatest part of them in their graves.

"The expectation of being relieved at the end of a twelvemonth, made this wretched life supportable; but unfortunately next season the mortality was so great among the soldiers sent from Senegal to relieve them, that not above three or four reached the fort alive, the rest died on their passage up the river. It was therefore impossible for the poor remains of the former garrison to quit it, and they were obliged to drag out in it another year, yet more tedious than the former; when the very few who remained alive were sent back to Senegal with ruined constitutions.

"Some miners were since sent to this place from England, in order to instruct the natives in a proper method of working their gold mines; but were all cut off, partly by the natives, but mostly by the climate."

In such an uncultivated swampy country, one could hardly expect a season of health. But even here our author maintains his position, that few places are universally healthy, and few so unhealthy as not to admit of periods and contracted spots, in which even newly arrived Europeans may live with enjoyment of health, and the other blessings of life. Though such as reside there, constantly retain throughout the whole year all the marks of broken constitutions; yet their diseases, during the dry months, are generally the remains of their former illness, contracted during the rainy seasons.

The author's *conjectures* on the causes of these diseases, being such as may be formed by a person less acquainted with the *facts*, we shall pass over to have room for more important matter. The properties of the Harmattan wind too, are also by this time pretty generally known. The medical directions are very important; but these, we presume, every practitioner who resorts to those regions, will study in the work itself; and more particularly the Treatise on the Diseases of Seamen, by the same writer.—This chapter concludes with a few remarks on the Canaries, Cape de Verd; the Islands of St. Thomas, Princess, Ferdinando Po, St. Helen's, Cape of Good Hope, Madagascar, Mascarenhas, Mauritius, and the Eastern shores of Africa.

The third chapter is on the unwholesome seasons and diseases in the East Indies. Here a succinct view is given of the English factories in Arabia, Persia, and also of the diseases which attend the uncultivated parts of Borneo, Sumatra, and Java, the coasts of Arracar,

Arracar, and Pegu, and the smaller Spice Islands, all of which have proved fatal to European traders or settlers. In all parts of the East Indies, near swamps, muddy banks, and foul shores, the same danger attends Europeans. In one place, near Indrapour, in Sumatra, no European can venture to sleep a night on shore.

After a short sketch of the Asiatic continent, the author proceeds to take a general survey of the state of health in the different European settlements in that part of the world. We extract the following passage, because it relates to a subject which is insisted upon by every practitioner in the tropical regions, but entirely overlooked or disbelieved in Great Britain. If the changes of the moon are really productive of these effects in the warmer regions, we can hardly conceive that they should be totally indifferent in these. The agues in our own island continue obstinate, even at this time, and afford a fair subject for investigation among the practitioners along the eastern coast.

" Bengal, says our author, next to Bencoolen, of all the English factories, proves the most fatal to Europeans. The rainy season commences at Bengal in June, and continues till October: the remainder of the year is healthy and pleasant. During the rains, this rich and fertile country is almost quite covered by the overflowing of the river Ganges, and converted as it were into a large pool of water. Diseases rage among the Europeans in the months of July, August, September, and October, attacking chiefly such as are lately arrived. Here, as in all other places, sickness is more frequent and fatal in some years than others. The distempers are fevers of the remitting or intermitting kind; sometimes they may begin under a continued form, and remain several days without any perceptible remission, but they have in general a great tendency to a remission. They are commonly accompanied with violent fits of rigors or shiverings, and with discharges of bile upwards and downwards. If the season be very sickly, some are seized with a malignant fever, of which they soon die: the body is covered with blotches of a livid colour, and the corpse in a few hours turns quite black and corrupted. At this time fluxes prevail, which may be called bilious or putrid, the better to distinguish them from others which are accompanied with an inflammation of the bowels. In all those diseases at Bengal, the lancet is cautiously to be used.

" It is a common observation, both at Bengal and Bencoolen, that the moon or tides have a remarkable influence there on intermitting fevers. I have been informed by a gentleman of undoubted veracity, and of great knowledge in medicine, that in fevers at Bengal, he could foretel the precise time when the patient would expire, it being generally about the hour of low water.

" Thus much is certain, that in the year 1762, after a great sickness, of which it was computed 30,000 blacks and 800 Europeans died in the province of Bengal, upon an eclipse of the moon, the English merchants and others, who had left off taking the bark, suffered a relapse. The return of this fever was so general on the day day of the eclipse, that there was not the least reason to doubt of the effect. However the moon's influence may operate, these observations furnish a useful hint, which is, in such situations, to take doses of bark at the full and change of the moon, as being the seasons found there to be most dangerous for an attack or relapse into those intermitting fevers."

The account of the other settlements follows, and a very interesting medical history of a Journey by land from India to Europe, by Mr. Ives.

The fourth chapter is on the most unwholesome seasons in the West Indies, and the diseases there prevalent. This commences with a comparative view of the health in the English, French, Dutch, and Spanish settlements. An account follows of the diseases of the southern part of the western hemisphere. The yellow fever is described in all the strong colours it exhibits; and our author shows that if the fever at Cadiz was not the same, it will not be easy to mark the difference: a dreary summary follows of the mortality during some memorable seiges and expeditions.

The second part contains advice for the preservation of Europeans resident in warm climates near the sea. This is, perhaps, the most valuable part of a work, the whole of which does credit to the diligence and abilities of the author. The first section gives a philosophical and interesting account of the signs by which an unhealthy situation is to be discovered; the second is on the employments which prove most fatal to Europeans in hot and unwholesome climates. This is a subject which cannot be too much or too often impressed on government, or the commanders of our armies and fleets. How many brave men might have been saved in the West Indies, if gangs of negroes had been hired to cut wood or bring water from places, to which men of that complexion may be exposed with little danger, compared to the fresh arrived European? A section follows, on the diseases incident to strangers in different climates; the seasons at which they occur, and to which they are confined; the manner in which European constitutions are seasoned to hot climates, and the necessity of removing during the sickly seasons.

" Strangers, says our author, should always leave unhealthy spots, for a few months during the sickly season, until they become well inured to the climate.

" This removal to a small distance from the seat of sickness, promises a security at least equally certain with the method now taken by Europeans, of shutting themselves up in their houses, and having no communication with the natives, during the rage of the plague in Turkey. It is likewise a precaution, upon which the safety of strangers, in unhealthy climates, may sometimes alone depend.

" One cannot, without astonishment, reflect on the blindness of mankind, in never discovering this so simple and easy a method, which their own observation must have every day pointed out to them:

them: yet our factories abroad have never paid any attention to it, and a proper method of doing it has never been recommended to them."

The following section, containing directions for providing a safe and convenient retreat during the unhealthy seasons, is full of valuable and practical remarks. A very ingenious proposal is annexed, to form floating factories at these periods, in such places as do not afford sufficient elevation by land for security from pestilential vapours or blasts. This, we are informed, has been sometimes attempted with success on the coast of Guinea.

Not less valuable are our authors remarks on the necessity of leaving an impure air after the attack of fever has commenced. Without this, he justly observes, we can expect no security from relapses or a long valetudinary state. What is principally insisted on here, is the safety with which a person may be removed in any state of a disease which arises from impure and pestilential atmosphere. Of many thousand patients, says Dr. Lind, whom I have visited in Haslar Hospital, for twenty-five years, nine-tenths were moved during the continuance of the fever, either from Spithead, from the ships in the harbour, or from the Marine Infirmary at Portsmouth; though they were brought by every different mode of conveyance, yet instead of suffering any injury by such transportation, under the most malignant symptoms of the disease, many received the greatest benefit.

A section follows, on the effect of a bad air on persons in health, as well as on those who are previously diseased: and on the consequences of removing the sick in fevers, from a bad to a good air;—with an inquiry how long the effects of bad air lie concealed in the body, and how far the fever thence produced, is contagious. On this last and most important subject, we cannot refrain from a few remarks. The first is one which we often have occasion to make when reviewing the performances of men of such acknowledged merit as Dr. Lind; viz. a wish that they would trust to the suggestions of their own minds, instead of attending to the inferences of others. Every fact is unquestionably valuable, but an inference is no otherwise valuable, than as it is well supported.

"How far, says our author, this fever, as produced by the land air, is contagious, it is more difficult to determine; the exemption from it, which those generally enjoy who sleep at a distance from the shore, while others, who have suffered by a neglect of that precaution are sick on board, seems to prove it void of any contagion. But, upon a more narrow examination, we shall be inclined to adopt a contrary opinion. Passing in silence the many means whereby infection may be communicated, so as to elude the strictest inquiry, not only immediately from the diseased person, but from his clothes or attendants, we need insist only on positive facts.

"In the Weazel and Hound, two sloops of war, which happened to be in the river Gambia, in the beginning of August, 1769, at which time the rainy season commenced that year, it was observed, the sickness did not begin till two or three days after they had had received an infected person on board, notwithstanding they had been eight or ten days in that river. Their being at the same time exposed to the land air, almost equally with those who were in the fort, by anchoring up the river, might greatly increase the malignity of the disease; but does not weaken the opinion of its being received by contagion, which is fully proved by a curious particular given by Mr. Robertson, in an account of his own case.

"This gentleman, when feeling the pulse of a boy dying in the fever, immediately as he expired, received a shock as though electrified, attended with a disagreeable sensation, not easily to be expressed, and quickly followed by a prostration both of strength and spirits, so that he had almost fainted before he reached his apartment; and afterwards suffered a very severe attack of the fever."

Now if Mr. Robertson and his patient were exposed to the same cause, what can be more probable than that they were both affected by that cause. It cannot surely be any argument in favour of contagion, that Mr. R. was seized so instantaneously, because so sudden an effect from contagion is, to say the least, much less probable than that he should feel at that moment, the first symptoms of a fever from a pestilential atmosphere.—The succeeding account of contagion on board the Merlin, appears to us easily accounted for by the ship fever being generated from the number of sick on board. We are aware that the author does not entirely overlook such a cause. It is therefore not our wish to accuse Dr. Lind of inattention, but of diffidence in not trusting to those numerous facts which have passed under his own observation.

The eighth section of this chapter, contains some remarks on the benefit of sea air in fevers, particularly instancing the advantage experienced from it in an epidemic fever in Naples. A very useful hint follows, on the construction of an infirmary on board a ship, to receive the sick of a factory during an unhealthy season. On the subject of seasoning European constitutions to warm climates, the remarks are particularly judicious.

Such are the principal cautions for Europeans residing near the sea coast in hot climates. A chapter follows of the means of preserving such as reside in more inland situations.

The third part comprehends advice to Europeans under the effect of the various diseases to which they in particular are subject in warm climates.—These are fever, dysentery, cholera, dry belly-ache, tetanus, and the barbiers, a species of palsy sometimes subsequent on exposure to the night air under intoxication in the East Indies.—Some directions follow for Europeans on their return home after having suffered from the warm climates: the whole concludes with an appendix on intermittent fevers, and on an easy way of rendering sea water fresh, and of preventing a want of provisions at sea.

Such is this work, which it is quite unnecessary for us to recommend. We have, however, thought it right, at these times, to acquaint our readers with some of its valuable co tents, which is all that that can be necessary to induce them to peruse the whole with diligence and frequency.

---

*Practical Observations on the Nature and Cure of Strictures in the Urethra.* By William Wadd, Member of the Royal College of Surgeons of London, 8vo. Callow.

It is now so long since we have heard of any mode of treating strictures unconnected with caustic, that we felt particular pleasure at receiving the work before us. It is true we are not without books on strictures, which explain the mode of treatment by the simple bougie; but they were written before Messrs. Home and others had so entirely established the fame of the caustic as to leave no other dispute, excepting concerning the composition of their destructive materials.

That the caustic is sometimes necessary cannot be questioned, that it is often unattended with ill consequences must be admitted, or it could not so long have maintained its ground. But it may fairly be doubted, whether it is as often necessary as employed; nor is it less certain, that instances have occurred, in which it has proved alarming, and even fatal. A work, therefore, which contains a general view of the circumstances under which each mode has been introduced, and practical inferences from impartial observation on the effects of each, seems, at present, particularly well timed.

The author begins by remarking, that immediately before the period in which Mr. Hunter flourished, the caustic had been by common consent proscribed. That gentleman, "whose life and writings make an important æra in surgery," revived its use. He recommended it, however, in a limited degree, and applied it with great caution in those cases only for which the common bougie was found insufficient.

"It is, says our author, at the points where the urethra is supposed to have the greatest degree of contraction, that strictures are most commonly found, viz. at four inches and a half, and at six and a half, from the external orifice. They are seldom found beyond this. Mr. Hunter says, he never saw a stricture in that part of the urethra which passes through the prostrate gland. Mr. Abernethy, however, has met with cases where all the symptoms remained, arising from a stricture at the neck of the bladder.

"The contractile power of the urethra, when in a healthy state, is a gentle and uniform action of its fibres; but when too frequently repeated by the excitement of any stimuli, the action becomes spasmodic, and may produce the disease in question.

"The causes of contractions of the urethra, has been a subject of general discussion. We are led to believe, that in certain constitutions there is a greater disposition to the formation of strictures than in others; where this happens, it is easy to conceive that occasional attacks of spasm, arising from any source, may produce this disease. The most commonly implied cause was neglected or mismanaged gonorrhœa. Mr. Hunter, however, has taught us to think differently. Nor does gonorrhœa seem instrumental in producing ducing strictures, excepting in common with other inflammation; —the seat of gonorrhœa and of stricture being rarely found the same. The formation of strictures has also been attributed to astringent injections used in the cure of gonorrhœa; on which account Mr. Home has, we find, entirely given them up, rather than incur a risk, however small it may be, of producing so seriously distressing a complaint. This does not accord with the general practice, nor with what Mr. Home afterwards observes of the natives of India, three-fourths of whom, of any rank, we are informed, are troubled with strictures, attributed to the effects of gonorrhœa, for which no local applications are used. Sir George Baker observes, 'It is generally to be wished, that all the injections commonly ordered for disorders of the urethra were gone into disuse; since every day's experience testifies, that such injudicious applications are, in the event, frequently the parent of obstinate and incurable obstructions of that passage.' Various other causes have been assigned, as the gravel and stone. They have also been known to arise from piles, and in some cases, from the temporary irritation of the parts following the use of a blister.

"Strictures are varied in their form and structure; those most commonly met with, are small ridges projecting into the canal, and forming a half circle; sometimes they are continued and form a circular band, not more in breadth, than if the part had been surrounded with a piece of packthread. Some are contracted for above an inch in length, which Mr. Home accounts for, by two strictures forming within an inch of each other, and the space between them becoming narrower than the rest of the canal. This may happen, but I have seen a thickening and contraction of the urethra to this extent. Sometimes two or more strictures are on the same side, the other being perfectly smooth. Very frequently there is a gradual contraction of the urethra to and from the stricture, so as to form a cone in the anterior and posterior part of it. Mr. Cooper, in 'First Lines of the Practice of Surgery,' describes the shape as resembling two long cones, with their points in contact. He adds, 'I am perfectly convinced that this is the case which frequently baffles the operation of caustic, and which receives most benefit from the employment of the common bougie."

A few observations follow, on the uncertain opinio ns of the ancient writers, concerning these subjects, with a short statement of the progress of the disease itself. After which, we have an historical account of the practice with caustic from the time Mr. Hunter first revived it, and at the same time gave the world a more rational account of the nature of strictures. Mr. Home and Mr. Whately are chiefly noticed, whose writings, well known to our readers, are shown not to be in every part perfectly consistent with themselves. Some remarks are interspersed on Dr. Andrews. The whole of this part is so entirely divested of all the acrimony of controversy, that we cannot help being pleased with the prospect of seeing medical disputations conducted with that politeness which should distinguish the professors of a liberal art. The different practice practice pursued by Mr. Home and Mr. Wheatly is accurately pointed out, and the inference follows from each, that whatever may be the application, a caustic can only act by destroying the part.

The principal objections against the caustic are stated with much candour, nor are its advantages undervalued. The application from the whole is, that where the common bougie will pass with sufficient ease to dilate the parts, the prospect of a more perfect or permanent relief is by no means sufficiently ascertained to authorise so violent and uncertain a remedy.

" As the difficulties, says our author, attending the introduction of the caustic into a passage so peculiarly constructed, were obvious to Mr. Hunter, he did not venture to revive the old practice without some modifications, and what he considered improvements. He adopted the use of a silver canula, first suggested by Wiseman, but its unyielding materials were found ill adapted to the flexible canal of the urethra; and that the middle of the stricture, the part intended to be destroyed, was frequently left untouched, whilst only the angle, between the stricture and the side of the urethra, was exposed to the caustic. These objections, we are informed by Mr. Home, did not escape Mr. Hunter's penetration. He not only saw them, but devised a mode, by which they might be removed. This improved method was the armed bougie. The account given of it is as follows:—' Take a bougie, of a size that can readily be passed down to the stricture, and insert a small piece of lunar caustic, but surrounding it every where laterally by the substance of the bougie.' This Mr. Home considers a very valuable instrument, on account of the facility and safety, with which it conveys the caustic to the stricture; and, indeed, he adds, ' the *quickness* with which it passes, prevents any injury to the membrane, where it is accidently brought to oppose it!'

" There are, however, objections made to this instrument by those who think, that the facility with which a bougie passes, depends upon the roundness of its point, and the smoothness of its surface. Some have even gone so far, as to think it the worst instrument for the purpose that could have been devised. This is the opinion of Mr. Whately, whose work I shall notice hereafter, who, as a great advocate for the caustic practice, ought to be attended to in this instance. His observations are strong, and to the point:—' For my own part,' he says, ' I think that a worse mode of applying lunar caustic to a stricture, in the curved part of the urethra, could not have been invented; every one who has had much practice in passing a bougie into the urethra, knows, that unless *its point be round*, and the whole bougie smooth, it passes along this canal with great difficulty, and not without giving pain. As, therefore, the extremity of a bougie, thus prepared, cannot be made round, I consider it an instrument very unfit for the purpose; to say nothing of the caustic stone, dropping from the bougie while in the urethra.' "

( To be concluded in our next. )

